# Clinicopathologic and prognostic significance of VEGF, JAK2 and STAT3 in patients with nasopharyngeal carcinoma

**DOI:** 10.1186/s12935-018-0605-0

**Published:** 2018-08-13

**Authors:** Jin-Zhang Cheng, Jun-Jun Chen, Kai Xue, Zong-Gui Wang, Dan Yu

**Affiliations:** 1 Department of Otolaryngology Head and Neck Surgery, the Second Hospital of Jilin University, No. 218, Ziqiang Street, Nanguan District, Changchun, 130041 Jilin Province People’s Republic of China; 2 Department of Pharmacy, the Second Hospital of Jilin University, Changchun, 130041 People’s Republic of China

**Keywords:** Nasopharyngeal carcinoma, Janus kinase 2, Signal transducer and activation of transcription 3, Vascular endothelial growth factor, Clinicopathologic characteristics, Prognosis

## Abstract

**Background:**

The aim of the study was to investigate the effect associated with the protein expression of VEGF, JAK2 and STAT3 on the clinicopathologic characteristics and prognosis in the development and progression of nasopharyngeal carcinoma (NPC).

**Methods:**

Fifty NPC patients in addition to 20 patients with chronic nasopharyngitis (CNP) were recruited for the purposes of the study. Western blotting and immunohistochemistry methods were employed to evaluate the protein expressions of JAK2, STAT3 and VEGF in the NPC and CNP tissues, with their respective correlations with the clinicopathologic characteristics of NPC patients subsequently analyzed. Spearman’s rank correlation coefficient and Kaplan–Meier method were conducted to evaluate the respective correlations of JAK2, STAT3 and VEGF with NPC as well as the survival rates of patients with NPC. Cox regression analyses was performed in determine the prognostic NPC factors.

**Results:**

Compared with the CNP tissues, the NPC tissues exhibited elevated levels of JAK2, STAT3 and VEGF which were subsequently determined to share a positive correlation with T stages, lymph node metastasis (LNM), N stages and clinical stages, while a negative correlation with survival rates were observed in the NPC patients. Positive correlations between the expressions of JAK2, STAT3 and VEGF were detected among the NPC tissues. NPC patients survival time with negative expressions of JAK2, STAT3 and VEGF were observed to be longer than that of NPC patients with positive expressions of JAK2, STAT3 and VEGF. T stage, LNM, N stage, clinical stage. The expressions of JAK2, STAT3 and VEGF were discovered to be independent risk factors associated with the prognosis of patients with NPC.

**Conclusion:**

The results obtained from the present study support the notion that higher expressions of JAK2, STAT3 and VEGF may be correlated with the clinicopathologic characteristics and prognosis of patients suffering from NPC.

## Background

Nasopharyngeal carcinoma (NPC) is a malignancy with a distinct ethnic and geographical distribution. NPC is known as a prevailing tumor commonly seen in the south of China as well as in Southeast Asia, especially in the Cantonese population, where its incidence has remained high for decades [1]. NPC in comparison to other head and neck (H&N) cancers, is characterized by a distinctive epidemiology, symptoms, biological markers, carcinogenic risk factors, and prognostic elements [2]. Although various etiological studies have highlighted a close correlation between NPC and multiple factors, such as living condition, genetics, viral infection, as well as environmental factors, the finer etiological factors on a molecular level are yet to be fully identified [3]. Viral infections, multiple somatic genetic as well as epigenetic changes have all been reported to synergistically disrupt normal cell function, playing a contributory role in the pathogenesis of NPC [4]. NPC is a relatively radiosensitive disease, some NPC patients suffer from distant metastases and local recurrences after radiotherapy due to radio resistance, with the greater majority of these patients eventually falling victim to metastasis and recurrence within one and half years of treatment [5].

Uncovering the associated signaling pathways involved with the finer biological mechanisms of NPC is an urgent requirement for improved targeted therapy for the disease [5]. Overexpressed epidermal growth factor receptor (EGFR) has been widely reported to be commonly observed in cases of NPC [6]. What’s more, overexpressed EGFR in primary tumors has been linked with tumor recurrence, metastasis, and poor survival in patients suffering from NPC [7]. Previous studies have suggested that EGFR plays a vital role in the development and progression of NPC [5]. While several biomarkers have been employed in the determination of the prognosis and metastasis of NPC patients, the molecular mechanisms regarding the development and progression of NPC remain largely unknown.

Signal activator and transducer of transcription 3 (STAT3) is an oncogenic transcription factor that often exists in many tissues, playing a prominent role in the regulation of cellular activity [8]. The overexpression of STAT3 has been observed among various types of cancers, with reports suggesting that overexpressed STAT3 promotes tumor genesis by protecting against cell apoptosis and enhancing angiogenesis, proliferation, invasion and metastasis [9]. Generally speaking, the activation of STAT3 signaling is strictly regulated and is associated with adverse cellular transformation [10]. Moreover, STAT3 is a critical transcription activator biomarker in tumor therapy and is also involved in a series of fundamental events implicated in the development of tumors [11]. Thus, examining the levels of STAT3 in colorectal tumors might be a significant indicator of the severity of prognosis and malignancy, and may possibly present a new target for the treatment of cancer.

Janus kinases (JAKs) are non-receptor tyrosine kinases involved in upstream intracellular signaling pathways that are subsequently activated following extracellular ligand binding to all kinds of cytokine and growth-factor receptors [11]. Janus kinase 2 gene (JAK2), a crucial member of the JAKs family, is located at the upstream of STAT3 signaling pathway and is the major activator of STAT3 [8]. Numerous studies have suggested that the carcinogenicity of JAK2 can lead to the constitutive activation of the JAK2/STAT3 signaling pathway [11]. The JAK/STAT pathway plays a crucial role in cytokine signaling cascades, which is also involved in multiple cellular functions such as differentiation, survival, proliferation and apoptosis [12, 13]. In addition, STAT3 possesses regulatory abilities in angiogenesis through the transcription of vascular endothelial growth factor (VEGF) [14]. VEGF is a potent angiogenic factor which plays a key role in a vast array of pathological processes [15]. Not only can VEGF act to enhance microvascular permeability, by possibly introducing tumor cell penetration into circulation [11], but also serves as an important mediator of tumor-induced angiogenesis, representing a potential target for anticancer therapy [16].

In regard to the above explored literature, during the present study we were of the opinion that the expressions of STAT3, JAK2 and VEGF were associated with proliferation, anti-apoptosis, and transformation in relation to the activation of human tumors to some extent. Hence, the current study was designed with the objective of investigating the association of STAT3, JAK2 and VEGF with the development and progression of NPC.

## Materials and methods

### Study subjects

Between February 2005 and November 2010, 103 NPC patients at the Second Hospital of Jilin University (62 males and 41 females; age 18–75 years; mean age: 50.8 ± 10.0 years) with confirmed pathological cases and diagnoses of non-keratinizing squamous cell carcinoma (SCC) (72 cases of differentiated and 31 cases of undifferentiated) were enrolled into the current study. Based on the 2003 revision of tumor node metastasis (TNM) staging system, which was made by the Union Internationale Contre le Cancer (UICC) and the American Joint Committee on Cancer (AJCC) [17], the primary tumors of the 50 NPC patents in the study were staged as follow: T_1_, 12 cases; T_2_, 10 cases; T_3_, 30 cases; T_4_, 51 cases; N_0_, 28 cases; N_1_, 12 cases; N_2_, 19 cases; N_3_, 44 cases; clinical stage I, 7 cases; II, 41 cases; III, 29 cases; IV, 26 cases; lymph node metastasis (LNM), 60 cases; non-LNM, 43 cases. The inclusion criteria were as follows: (1) patients who were diagnosed with NPC and had undergone surgical treatment at the Oncology Department of the Second Hospital of Jilin University, (2) patients with adequate paraffin embedded NPC tissue specimens in connection with the Department of Pathology, with complete clinical data and effective contact methods. The exclusion criteria were as follows: (1) patients who underwent surgery at the Second Hospital of Jilin University with previous NPC biopsy collected at another hospital, (2) patients that had received neoadjuvant chemotherapy, neoadjuvant targeted therapy and immunotherapy prior to surgery. The nasopharyngeal mucosa specimens from 20 patients were subsequently (46 males and 32 females; age 21–71 years; mean age: 50.1 ± 7.9 years) pathologically confirmed to be cases of chronic nasopharyngitis (CNP) and were regarded to be the control group. All patients enrolled in the study signed informed consent documentation. All experimental procedures of the study were conducted under the approval of the Ethics Committee of the Second Hospital of Jilin University.

### Immunohistochemical staining

Fifty tissue specimens from NPC patients in addition to 20 tissue specimens from CNP patients were collected, followed by the application of immunohistochemical staining. The samples were fixed with formalin and embedded with paraffin (for the Department of Pathology collection purposes). The paraffin-embedded tissue samples were subsequently cut into 6–8 sections (slice thickness: 3 μl) continuously. After being placed in antistripping agent at 65 °C for 2 h, the tissue sections were deparaffinized in xylene, hydrated in gradient ethanol, and rinsed 3 times using distilled water (2 min/time). The sections were then cooked with Tris–ethylenediaminetetraacetic acid (Tris–EDTA) buffer (pH 9.0, Wuhan Boster Biological Technology, Ltd., Wuhan, China) under high-pressure conditions and immersed in 3% hydrogen peroxide (H_2_O_2_) solution at room temperature for 10 min, followed by rinsing with phosphate buffer saline (PBS) solution (Wuhan Boster Biological Technology, Ltd., Wuhan, China). Next, normal goat serum was added to the sections for incubation at 37 °C for 10 min, followed by the addition of rabbit anti-human JAK2, rabbit-antihuman STAT3 and rabbit anti-human VEGF, respectively in a 4 °C refrigerator for 24 h (1:100 dilution) for incubation purposes. The sections were then rinsed with distilled water; followed by the addition of the secondary antibody rabbit anti-mouse working solution (Wuhan Boster Biological Technology, Ltd., China) and incubated at 37 °C for 30 min. After staining with diaminobenzidine (DAB), the sections were counterstained using hematoxylin (Wuhan Boster Biological Technology, Ltd., Wuhan, China) for 1–2 min, dehydrated with graded ethanol, vitrified with xylene and sealed with neutral gum.

The sections were then rinsed with phosphate buffer saline (PBS) (3 × 15 min) (Wuhan Boster Biological Technology, Ltd., China) after the end of each procedure, followed by the analysis of the staining results. The control sections were treated with PBS in lieu of the initial antibody. The immunohistochemical staining of JAK2, STAT3, and VEGF was represented by a brownish yellow or brown color, which was considered to be positive. On the basis of the cell pigmenting degree, the expressions of JAK2, STAT3 and VEGF were classified into three grades: 1 point (+); 2 points (+ +); 3 points (+ + +). The markers of “+”, “+ +”, “+ + +” were respectively regarded as weakly positive, moderately positive and strongly positive. The number of positive cells, less than 25% of the total number of positive cells was considered to be weakly positive (1 point); while 25–49% was moderately positive (2 points) and 50% or more was regarded as strongly positive (3 points). The average percentage of positive cells among 100 cells from 5 visions using a high powered microscope (400 ×) were counted, and the results were expressed as a percentage between 0 and 100%. The grades based on the product of the percentage of positive cell were: negative and staining intensity (0–3 points), positive (> 3 points) [18–20].

### Western blotting

The total cell protein of the sections was extracted; followed by determination of the protein concentration using a bovine serum albumin (BSA) protein assay (Wuhan Boster Biological Technology, Ltd., Wuhan, China). Sample buffer was added to the proteins and boiled for 10 min at 95 °C. More specifically, the proteins were subjected to electrophoresis methods and isolated using 10% sodium dodecyl sulfate–polyacrylamide gel electrophoresis (SDS-PAGE) gel (Electrophoresis voltage: 80–120 V), then transferred onto polyvinylidene fluoride (PVDF) (Voltage: 100 mV, Time: 45–70 min). After blockade with 5% bovine serum albumin (BSA) for 1 h, the membranes were bottled with primary antibodies JAK2 (1:1000; Abcam Inc., Cambridge, MA, USA), STAT3 (1:1000, Abcam Inc., Cambridge, MA, USA) and VEGF (1:1000, Abcam Inc., Cambridge, MA, USA), at 4 °C overnight, then washed 3 times with TBST (5 min per wash) and added with secondary antibodies at room temperature for 1 h. The membranes were rinsed with PBS and developed by using Erythrina cristagalli lectin (ECL) reagents. Glyceraldehyde-3-phosphate dehydrogenase (GAPDH) (1:5000, Shanghai Kang Chen Bio-tech Co., Ltd., China) was considered as the internal control for adjustment purposes. Images were acquired using a Gel Doc EZ Imager (Bio-Rad Laboratories, Inc. California, USA) and the gray level of target protein band was analyzed using Image J software.

### Follow-up

After discharge, all patients who underwent surgery were prospectively monitored by means of clinic visits or follow-up by telephone. The follow up process ended in November 2015. The shortest survival time was 8 months post operation, the longest was 60 months, and the average survival time was 32.6 months.

### Statistical analysis

All statistical analyses were performed with the SPSS 19.0 software (IBM Corp., Armonk, NY, USA). Continuous data were displayed as mean ± standard deviation (SD), whereby the differences between two groups were analyzed by *t* test and paired t-test. Categorical data were expressed as a ratio or percentage, while the differences among groups were compared by Chi square test. The level of significant difference was set at *P* < 0.05. The correlations between JAK2, STAT3 and VEGF were analyzed using Spearman’s rank correlation coefficient. The correlation of JAK2, STAT3 and VEGF with the survival rates of the positive and negative NPC patients were evaluated using the Kaplan–Meier method. The NPC patient prognostic factors were identified by Cox regression analyses.

## Results

### JAK2, STAT3 and VEGF expressed at a high level in NPC tissues

**Fig. 1:**
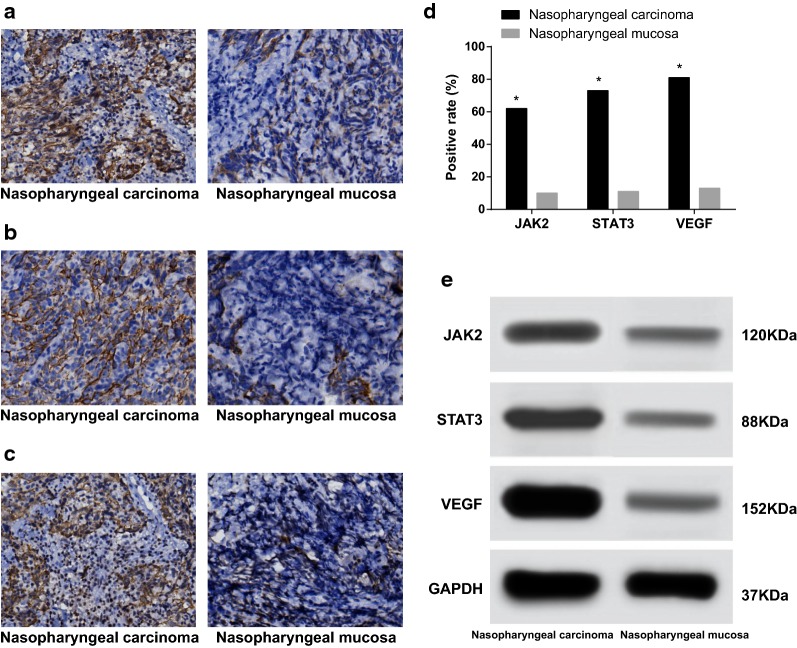
Protein expression of JAK2, STAT3 and VEGF in the NPC and CNP tissues; **a** expression of JAK2 protein; **b** expression of STAT3 protein; **c** expression of VEGF protein; **d** the positive expression rate of JAK2, STAT3 and VEGF; **e** gray value of JAK2, STAT3 and VEGF proteins; *NPC* nasopharyngeal carcinoma, *CNP* chronic inflammation of nasopharyngeal, *JAK2* janus kinase 2, *STAT3* signal transducer and activator of transcription-3, *VEGF* vascular endothelial growth factor

The immunohistochemical staining results revealed that the positive expression of JAK2 in NPC tissues were predominately localized in the cytoplasm with dark brown color, however very low levels were expressed in the CNP tissues (Fig. 1a). The positive expression of STAT3 in NPC tissues mainly exhibited in the cytoplasm and rarely in the capsule with dark brown, while meagre staining was observed in CNP tissues (Fig. 1b). The stained VEGF in NPC tissues were generally observed in the cytoplasm with dark brown or brown particles, while no distinct staining was observed in the CNP tissues (Fig. 1c). Among the 103 NPC tissues, the positive expressions of JAK2, STAT3 and VEGF were noted in 62 cases (60.2%), 73 cases (70.9%) and 81 cases (78.6%) respectively. Regarding the 78 CNP tissues, the positive expressions of JAK2, STAT3 and VEGF were observed in 10 cases (12.8%), 11 cases (14.1%) and 13 cases (16.7%), which indicated that the expressions of JAK2, STAT3 and VEGF in the NPC tissues were significantly higher than those in the CNP tissues (all *P* < 0.05) (Fig. 1d). The Western blotting results obtained indicated that the protein expressions of JAK2, STAT3 and VEGF among the NPC tissues were significantly higher than those in CNP tissues (all *P* < 0.05) (Fig. 1e).

### JAK2, STAT3 and VEGF were positively associated with T stage, LNM, N stage and clinical stage

The protein expressions of JAK2, STAT3 and VEGF in the NPC tissues were found to share a positive association with the T stage, LNM, N stage and clinical stage (all *P* < 0.05); however, no significant differences were detected in relation to the expression of JAK2, STAT3 and VEGF proteins among NPC patients with different age, gender, differentiation type, smoking and family history (all *P* > 0.05) (Table 1).

**Table 1:** Correlation of positive and negative expressions of JAK2, STAT3 and VEGF with clinicopathologic characteristics of NPC

Clinicopathologic characteristics	Case	JAK2		χ^2^	*P*	STAT3		χ^2^	*P*	VEGF		χ^2^	*P*
		Positive	Negative			Positive	Negative			Positive	Negative		
Gender													
Male	62	38	24	0.295	0.587	44	18	0.220	0.639	44	18	5.459	0.622
Female	41	24	17			29	12			37	4		
Age (year)													
< 50	47	32	15	0.274	0.600	34	13	1.862	0.172	36	11	1.026	0.311
≥ 50	56	30	26			39	17			45	11		
T stage													
T1-2	22	12	10	0.373	0.042	11	11	3.311	0.015	15	7	1.822	0.002
T3-4	81	50	31			62	19			66	15		
LNM													
Yes	60	41	19	3.974	0.046	48	12	0.421	0.016	51	9	3.460	0.043
No	43	21	22			25	18			30	13		
N stage													
N0	28	13	15	0.704	0.015	25	3	0.317	0.033	26	2	4.627	0.031
N1-3	75	49	26			48	27			55	20		
Differentiation type													
Differentiated	72	46	26	1.363	0.243	50	22	0.211	0.646	56	16	0.039	0.843
Undifferentiated	31	16	15			23	8			25	6		
Clinical stage													
I–II	48	22	26	5.655	0.017	29	19	0.771	0.038	29	19	13.942	<0.001
III–IV	55	40	15			44	11			52	3		
Smoking history													
Yes	32	23	9	0.571	0.450	25	7	4.099	0.053	27	5	0.909	0.340
No	71	39	32			48	23			54	17		
Family history													
Yes	19	11	8	0.051	0.821	13	6	0.672	0.412	14	5	1.449	0.229
No	84	51	33			60	24			67	17		

*NPC* nasopharyngeal carcinoma, *LNM* lymph node metastasis, *JAK2* Janus kinase 2, *STAT3* Signal transducer and activator of transcription-3, *VEGF* Vascular endothelial growth factor, T stage, lymph node metastasis, N stage and clinical stage, all *P* < 0.05

### Interrelationships among the expression of JAK2, STAT3 and VEGF in NPC tissues

Among the 103 NPC cases, there were 62 and 54 cases with positive expression of JAK2 and STAT3 respectively. Furthermore, there were 41 cases with negative JAK2 expression in addition to 22 cases with negative STAT3 expression. Spearman’s rank correlation coefficient revealed that there was a positive association between the expressions of JAK2 and STAT3 in NPC tissues (R_s_ = 0.439, *P *= 0.001). Furthermore, there were 55 cases with positive VEGF, 62 cases with positive JAK2, 15 cases with negative VEGF as well as 41 cases with negative JAK2. Spearman’s rank correlation coefficient demonstrated that the expression of JAK2 and VEGF in the NPC tissues was positively related (R_s_ = 0.302, *P *= 0.002) (Table 2). There were 65 cases with positive expression of STAT3, 81 cases with positive VEGF, 11 cases with negative STAT3 as well as 22 cases with negative VEGF. The Spearman’s rank correlation coefficient indicated that the expression of VEGF and STAT3 in the NPC tissues shared a statistically positive correlation (R_s_ = 0.239, *P* = 0.0015) (Table 3).

**Table 2:** Interrelationships among the expressions of JAK2, STAT3 and VEGF in NPC tissues

JAK2	STAT3		Total	Spearman’s rank correlation coefficient	*P*	VEGF		Total	Spearman’s rank correlation coefficient	*P*
	Positive	Negative				Positive	Negative			
Positive	54	8	62	R_S_ = 0.439	< 0.001	55	7	62	R_S_ = 0.302	0.002
Negative	19	22	41			26	15	41		
Total	73	30	103			81	22	103		

*NPC* nasopharyngeal carcinoma, *JAK2* Janus kinase 2, *STAT3* Signal transducer and activator of transcription-3, *VEGF* Vascular endothelial growth factor

**Table 3:** Association between the expressions of STAT3 and VEGF in NPC tissues

VEGF	STAT3		Total	Spearman’s rank correlation coefficient	*P*
	Positive	Negative			
Positive	62	19	81	R_S_ = 0.239	0.015
Negative	11	11	22		
Total	73	30	103		

*NPC* nasopharyngeal carcinoma, *JAK2* Janus kinase 2, *STAT3* Signal transducer and activator of transcription-3, *VEGF* Vascular endothelial growth factor

### Correlation of the protein expression of JAK2, STAT3, VEGF with prognosis for patients with NPC

**Fig. 2:**
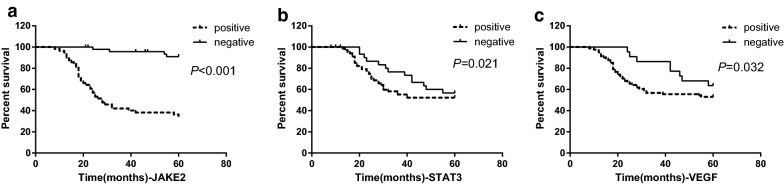
Kaplan-Meier survival curves for NPC patients with negative and positive expressions of JAK2 (**a**), STAT3 (**b**) and VEGF (**c**); *NPC* nasopharyngeal carcinoma; *JAK2* janus kinase 2, *STAT3* signal transducer and activator of transcription-3, *VEGF* vascular endothelial growth factor

The Kaplan–Meier survival curve results demonstrated that the survival time of NPC patients with negative expression of JAK2 was 58.7 ± 5.3 months, while the survival time of patients with positive JAK2 expression was 33.6 ± 19.7 months, which indicated that the survival time of the NPC patients with negative expression of JAK2 was significantly longer than that of NPC patients with positive JAK2 expressions (*P* < 0.05, Fig. 2a). The survival time of NPC patients with negative STAT3 expression was 53.6 ± 13.1 months, while the survival time of the patients with positive STAT3 expression was 39.5 ± 20.8 months, demonstrating that the survival time of NPC patients with negatively expressed STAT3 was longer than that of NPC patients with positive expressions of STAT3, a difference of which was determined to be statistically significant (*P* < 0.05, Fig. 2b). Furthermore, the survival time of NPC patients with negative VEGF expression was 52.0 ± 14.1 months, while the survival time of the patients with positive VEGF expression was 41.3 ± 20.7 months, suggesting that the survival time of NPC patients with negative VEGF expression was longer than that of NPC patients with positive VEGF expression, with the difference determined to be statistically significant (*P *< 0.01, Fig. 2c).

### Multivariate analysis of prognostic factors for NPC patients

Multivariate Cox regression analysis revealed that T stage, LNM, N stage, clinical stage and expression of JAK2, STAT3 and VEGF were independent risk factors associated with the prognosis of NPC patients (all *P *< 0.05), while the factors of gender, age, differentiation type, smoking and family history had no effect on the prognosis of patients with NPC (all *P* < 0.05) (Table 4).

**Table 4:** Results of multivariate analysis of prognostic factors for NPC patients

Factor (s)	B	SE	Wald	df	*P*	Exp (B)	95.0% CI for Exp (B)	
							Lower	Upper
Gender	0.102	0.354	0.083	1	0.774	1.107	0.553	2.218
Age	− 0.142	0.331	0.184	1	0.668	0.868	0.453	1.661
T stage	− 0.130	0.413	0.100	1	0.042	1.078	0.391	1.972
LNM	1.591	0.415	14.730	1	0.000	4.910	2.179	11.067
N stage	2.328	0.774	9.034	1	0.003	10.253	2.247	46.780
Differentiation type	0.006	0.371	0.000	1	0.988	1.0006	0.486	2.079
Clinical stage	3.247	0.608	28.516	1	0.000	25.701	7.807	84.615
Smoking	0.749	0.361	4.299	1	0.380	0.116	0.102	1.296
Family history	− 1.038	0.538	3.727	1	0.054	0.354	0.123	1.016
JAK2	1.583	0.461	11.812	1	0.001	4.868	1.974	12.006
STAT3	− 0.041	0.397	0.010	1	0.018	1.960	0.441	2.092
VEGF	− 0.820	0.609	1.815	1	0.017	1.440	0.133	1.453

*NPC* nasopharyngeal carcinoma, *LNM* lymph-node metastasis, *JAK2* Janus kinase 2, *STAT3* Signal transducer and activator of transcription-3, *VEGF* Vascular endothelial growth factor

## Discussion

NPC is widely acknowledged for its high-metastatic features in addition to its high rate of lymph node metastasis [21, 22]. During the present study fifty NPC patients as well as 20 patients with CNP were recruited in order to investigate the correlation of the JAK2/STAT3/VEGF pathway with the development and progression of NPC. We subsequently set out to examine the expressions of JAK2/STAT3/VEGF and their respective and collective correlations with the prognoses of NPC as well as the clinicopathologic characteristics.

Our findings revealed that the protein expressions of JAK2, STAT3 and VEGF in NPC tissues were markedly increased when compared to those in among the CNP tissues, with their expressions found to share a positive correlation with the T stage, LNM, N stage and clinical stage. STAT3 plays a primary role in relation to survival signaling and is habitual activation in transfected cells [23]. Various reports have suggested in various human tumors that the activation of STAT3 is associated with proliferation, cytokine-induced anti-apoptosis, and transformation [24]. Investigators have examined p-STAT3 expression among various malignancies such as gastric, renal, and ovarian cancers; squamous cell and hepatocellular carcinoma; and anaplastic large cell lymphoma [9]. Numerous reports have implicated the expression of p-STAT3 with lymph node spread as well as the depth of invasion in colorectal cancer [25]. Accumulating evidence has suggested that abnormalities in the JAK/STAT pathway can influence the oncogenesis of several cancers, while its inhibition has been shown to promote cancer cell growth and induce the apoptosis of tumor cells [26]. STAT3 has been speculated to be a factor in the regulation of angiogenesis through the modulation of VEGF (a key regulator of angiogenesis) [24]. VEGF can enhance microvascular permeability, potentially facilitating tumor cell penetration into the circulation [11]. Consistently, Jin et al. indicated that VEGF-C is a downstream target gene with the regulation of STAT3, while suggesting that it is a lymphatic growth stimulating factor, capable of regulating the differentiation and proliferation of lymphatic endothelial cells as well as accelerating tumor processes in LNM [10].

The Kaplan–Meier and Cox regression analyses results highlighted a negative correlation between the expressions of JAK2, STAT3 and VEGF and the survival of NPC patients. Besides, JAK2, STAT3 and VEGF were independent risk factors for prognosis of NPC patients. Reports have suggested that STAT3 is a key regulator of immunosuppression in addition to linking its expression with poor prognosis [9]. The abnormal activation of STAT3 is linked to tumor stage and prognosis in gastrointestinal tumors [25]. The activation of STAT3 has been linked to tumor development, primarily via its anti-apoptotic effects, immune escape, promotion of angiogenesis and poor prognosis [27, 28]. Accumulating evidence has implied that constitutive activation of the JAK/STAT pathway occurs frequently in diverse types of tumor cells and contributes to malignant progression [11]. In line with our study, recent evidence has also implicated that the JAK2/STAT3 signaling pathway from a regulatory perspective due to its involvement in various fundamental biological processes as well as the pathogenesis of cancer. The deregulation of the JAK2/STAT3 signaling pathway in particular, is frequently observed in primary tumors and can result in the increased survival of tumors and enhanced angiogenesis [29]. Besides, in cases of esophageal cancer, the expression of VEGF-C has been suggested to result in poor prognosis, in addition to being highlighted as an effective prediction of LNM [30]. Moreover, the expressions of VEGF-C in many tumors have been closely linked to sentinel LNM, lymphatic vessel invasion and distant metastasis in addition to being a predictor of poor prognosis [31].

## Conclusion

Taken together, the key findings of our study highlighted a relationship between the expressions of JAK2, STAT3 and VEGF and the development and progression of NPC. This finding may provide essential insight into the underlying mechanism in relation to the effects of the JAK2/STAT3/VEGF signaling pathway on the clinicopathologic characteristics and prognosis of NPC. However, there were certain limitations faced during our study; we did not identify the specific mechanism of each prognostic factor acknowledged in this study. Nevertheless, further studies with larger sample size will be conducted in order to strengthen our results and develop stronger clinical values.

## Abbreviations

NPC: nasopharyngeal carcinoma
CNP: chronic nasopharyngitis
LNM: lymph node metastasis
H&N: head and neck
EGFR: epidermal growth factor receptor
STAT3: signal activator and transducer of transcription 3
JAKs: janus kinases
JAK2: janus kinase 2 gene
VEGF: vascular endothelial growth factor
SCC: squamous cell carcinoma
TNM: tumor node metastasis
UICC: Union Internationale Contre le Cancer
AJCC: the American Joint Committee on Cancer
LNM: lymph node metastasis
Tris–EDTA: Tris–ethylenediaminetetraacetic acid
PBS: phosphate buffer saline
BSA: the bovine serum albumin
SDS-PAGE: sodium dodecyl sulfate–polyacrylamide gel electrophoresis
PVDF: polyvinylidene fluoride
ECL: erythrina cristagalli lectin
GAPDH: glyceraldehyde-3-phosphate dehydrogenase
SD: standard deviation

## References

[1] Wu RW, Chu ES, Yow CM, Chen JY (2006). Photodynamic effects on nasopharyngeal carcinoma (NPC) cells with 5-aminolevulinic acid or its hexyl ester. Cancer Lett.

[2] Adham M, Kurniawan AN, Muhtadi AI, Roezin A, Hermani B, Gondhowiardjo S (2012). Nasopharyngeal carcinoma in Indonesia: epidemiology, incidence, signs, and symptoms at presentation. Chin J Cancer.

[3] Thompson L (2005). Nasopharyngeal carcinoma. Ear Nose Throat J.

[4] Tao Q, Chan AT (2007). Nasopharyngeal carcinoma: molecular pathogenesis and therapeutic developments. Expert Rev Mol Med.

[5] Ruan L, Li XH, Wan XX, Yi H, Li C, Li MY (2011). Analysis of EGFR signaling pathway in nasopharyngeal carcinoma cells by quantitative phosphoproteomics. Proteome Sci.

[6] Tang CE, Guan YJ, Yi B, Li XH, Liang K, Zou HY (2010). Identification of the amyloid beta-protein precursor and cystatin C as novel epidermal growth factor receptor regulated secretory proteins in nasopharyngeal carcinoma by proteomics. J Proteome Res.

[7] Pan J, Kong L, Lin S, Chen G, Chen Q, Lu JJ (2008). The clinical significance of coexpression of cyclooxygenases-2, vascular endothelial growth factors, and epidermal growth factor receptor in nasopharyngeal carcinoma. Laryngoscope.

[8] Zhao M, Gao FH, Wang JY, Liu F, Yuan HH, Zhang WY (2011). JAK2/STAT3 signaling pathway activation mediates tumor angiogenesis by upregulation of VEGF and bFGF in non-small-cell lung cancer. Lung Cancer.

[9] Heimberger AB, Priebe W (2008). Small molecular inhibitors of p-STAT3: novel agents for treatment of primary and metastatic CNS cancers. Recent Pat CNS Drug Discov.

[10] Jin C, Wang A, Chen J, Liu X, Wang G (2012). Relationship between expression and prognostic ability of PTEN, STAT3 and VEGF-C in colorectal cancer. Exp Ther Med.

[11] Ji Y, Wang Z, Li Z, Li K, Le X, Zhang T (2012). Angiotensin II induces angiogenic factors production partly via AT1/JAK2/STAT3/SOCS3 signaling pathway in MHCC97H cells. Cell Physiol Biochem.

[12] Bromberg JF (2001). Activation of STAT proteins and growth control. BioEssays.

[13] Kisseleva T, Bhattacharya S, Braunstein J, Schindler CW (2002). Signaling through the JAK/STAT pathway, recent advances and future challenges. Gene.

[14] Gong W, Wang L, Yao JC, Ajani JA, Wei D, Aldape KD (2005). Expression of activated signal transducer and activator of transcription 3 predicts expression of vascular endothelial growth factor in and angiogenic phenotype of human gastric cancer. Clin Cancer Res.

[15] Banks RE, Forbes MA, Kinsey SE, Stanley A, Ingham E, Walters C (1998). Release of the angiogenic cytokine vascular endothelial growth factor (VEGF) from platelets: significance for VEGF measurements and cancer biology. Br J Cancer.

[16] Schlaeppi JM, Wood JM (1999). Targeting vascular endothelial growth factor (VEGF) for anti-tumor therapy, by anti-VEGF neutralizing monoclonal antibodies or by VEGF receptor tyrosine-kinase inhibitors. Cancer Metastasis Rev.

[17] Penault-Llorca F (2003). Comments on the new American Joint Committee on Cancer TNM staging for breast cancer. What’s new for the pathologist?. Ann Pathol.

[18] Laurinaviciene A, Dasevicius D, Ostapenko V, Jarmalaite S, Lazutka J, Laurinavicius A (2011). Membrane connectivity estimated by digital image analysis of HER2 immunohistochemistry is concordant with visual scoring and fluorescence in situ hybridization results: algorithm evaluation on breast cancer tissue microarrays. Diagn Pathol.

[19] Liu F, He Y, Cao Q, Liu N, Zhang W (2016). TBL1XR1 is highly expressed in gastric cancer and predicts poor prognosis. Dis Markers.

[20] Volm M, Koomagi R, Mattern J (1997). Prognostic value of vascular endothelial growth factor and its receptor Flt-1 in squamous cell lung cancer. Int J Cancer.

[21] You Y, Shan Y, Chen J, Yue H, You B, Shi S (2015). Matrix metalloproteinase 13-containing exosomes promote nasopharyngeal carcinoma metastasis. Cancer Sci.

[22] Hwang CF, Chien CY, Huang SC, Yin YF, Huang CC, Fang FM (2010). Fibulin-3 is associated with tumour progression and a poor prognosis in nasopharyngeal carcinomas and inhibits cell migration and invasion via suppressed AKT activity. J Pathol.

[23] Chen SH, Murphy DA, Lassoued W, Thurston G, Feldman MD, Lee WM (2008). Activated STAT3 is a mediator and biomarker of VEGF endothelial activation. Cancer Biol Ther.

[24] Rhee YH, Jeong SJ, Lee HJ, Lee HJ, Koh W, Jung JH (2012). Inhibition of STAT3 signaling and induction of SHP1 mediate antiangiogenic and antitumor activities of ergosterol peroxide in U266 multiple myeloma cells. BMC Cancer.

[25] Kusaba T, Nakayama T, Yamazumi K, Yakata Y, Yoshizaki A, Nagayasu T (2005). Expression of p-STAT3 in human colorectal adenocarcinoma and adenoma; correlation with clinicopathological factors. J Clin Pathol.

[26] Niwa Y, Kanda H, Shikauchi Y, Saiura A, Matsubara K, Kitagawa T (2005). Methylation silencing of SOCS-3 promotes cell growth and migration by enhancing JAK/STAT and FAK signalings in human hepatocellular carcinoma. Oncogene.

[27] Senft C, Priester M, Polacin M, Schroder K, Seifert V, Kogel D (2011). Inhibition of the JAK-2/STAT3 signaling pathway impedes the migratory and invasive potential of human glioblastoma cells. J Neurooncol.

[28] Kortylewski M, Kujawski M, Wang T, Wei S, Zhang S, Pilon-Thomas S (2005). Inhibiting Stat3 signaling in the hematopoietic system elicits multicomponent antitumor immunity. Nat Med.

[29] Buettner R, Mora LB, Jove R (2002). Activated STAT signaling in human tumors provides novel molecular targets for therapeutic intervention. Clin Cancer Res.

[30] Tanaka T, Ishiguro H, Kuwabara Y, Kimura M, Mitsui A, Katada T (2010). Vascular endothelial growth factor C (VEGF-C) in esophageal cancer correlates with lymph node metastasis and poor patient prognosis. J Exp Clin Cancer Res.

[31] Rinderknecht M, Detmar M (2008). Tumor lymphangiogenesis and melanoma metastasis. J Cell Physiol.

